# Supplementary material for: The arrangement of anisotropic spin couplings can optimize sensitivity of the cryptochrome radical pair to the direction of geomagnetic field
Source: Sci Rep. 2026 Jan 13;16:1961. doi: 10.1038/s41598-025-32180-x (PMC12805005; doi:10.1038/s41598-025-32180-x)
Supplement: Supplementary file 1 — Supplementary Information. [file 41598_2025_32180_MOESM1_ESM.pdf]

The arrangement of anisotropic spin couplings can optimize sensitivity of the cryptochrome radical pair to the direction of geomagnetic field

Victor Bezchastnov[1,*] and Tatiana Domratcheva[2]

[1]Max Planck Institute for Medical Research, Department of Biomolecular Mechanisms, Jahnstrasse 29, 69120 Heidelberg, Germany

[2]Lomonosov Moscow State University, Department of Chemistry, 119991 Moscow, Russia

*victor.beschastnov@mpimf-heidelberg.mpg.de

Supplementary Materials

Contents

1 The basic and expanded radical-pair models

2 Hyperfine couplings in the expanded models

3 The interconversion triplet yield in the basic and expanded models

1 The basic and expanded radical-pair models

The Supplementary Materials illustrate the anisotropic response to the direction of a 50 μT magnetic field at expanding the HFC contributions in the basic models RP1, RP3, and RP4 of the cryptochrome radical pair formed by the FAD and Trp radicals. The HFC in the basic models, which are described in Section 2 of the main text, is determined by the contributions of FAD N5 and Trp N1. In the expanded models, denoted below by the letters a, b, c, and d, we subsequently increase the number of the HFC contributions by taking into account the contributions of FAD N10 (a), FAD H8 (b), Trp H3a (c), and Trp H3b (d), so that the HFC in the models (d) is determined by a total of six terms. Figure S1 shows the positions of the nitrogens N5, N1, N10 and hydrogens H8, H3a, H3b, contributing to the expanding HFC, in the chemical structure of the radicals.

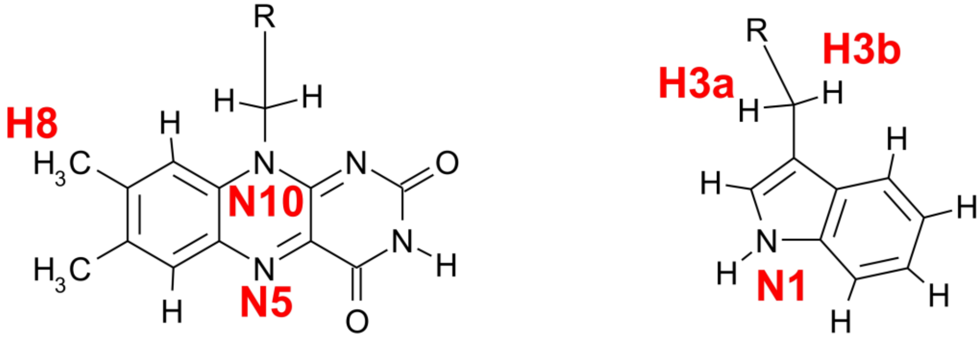

Figure S1. The structure of the FAD (left) and Trp (right) radicals, and the notations (in red colour) of the atoms contributing to the HFC in the basic and expanded models.

2 Hyperfine couplings in the expanded models

The HFC tensors included in the simulation of the magnetic response of the expanded models were computed from the electron spin densities same as those used to compute the FAD N5 and Trp N1 HFC tensors taken into account in the basic models. The additional tensors are represented below by the principal values in the units of mT, and the principal directions in the coordinate frame with the *xy*-plane defined by the HFC axes of the RP1 model.

FAD N10	$A_1 = 0.0037$	$\boldsymbol{e}_1 = (0.7360, -0.4780, -0.4794)$
	$A_2 = 0.0143$	$\boldsymbol{e}_2 = (0.3860, -0.2855, 0.8772)$
	$A_3 = 0.7410$	$\boldsymbol{e}_3 = (0.5562, 0.8307, 0.0026)$
FAD H8	$A_1 = 0.7150$	$\boldsymbol{e}_1 = (-0.3044, 0.0065, 0.9504)$
	$A_2 = 0.7254$	$\boldsymbol{e}_2 = (-0.8012, -0.5569, -0.2187)$
	$A_3 = 0.8116$	$\boldsymbol{e}_3 = (0.5151, -0.8280, 0.2213)$
Trp H3a	$A_1 = 0.0011$	$\boldsymbol{e}_1 = (0.2902, -0.9509, 0.1077)$
	$A_2 = 0.0016$	$\boldsymbol{e}_2 = (0.9499, 0.2999, 0.0087)$
	$A_3 = 0.1728$	$\boldsymbol{e}_3 = (-0.1156, 0.0077, 0.9903)$
Trp H3b	$A_1 = 0.8359$	$\boldsymbol{e}_1 = (0.2320, -0.9693, 0.0082)$
	$A_2 = 0.8644$	$\boldsymbol{e}_2 = (0.6223, 0.2125, 0.7534)$
	$A_3 = 0.9905$	$\boldsymbol{e}_3 = (-0.7476, -0.1239, 0.6525)$

Table S1. The principal values A_1 , A_2 , A_3 and the corresponding principal directions $\boldsymbol{e}_1$, $\boldsymbol{e}_2$, $\boldsymbol{e}_3$ of the HFC tensors accounted for in the expanded models of the radical pair.

3 The interconversion triplet yield in the basic and expanded models

Figures S2—S6 compare the triplet interconversion yield for the basic and expanded models of the cryptochrome radical pair. The plots show the dependence of the yield on the either azimuthal or polar angle defining the magnetic field direction at the fixed values of the other angle, as indicated in each plot. For the RP1 model and its expansions, the angles (φ and ϑ) refer to the coordinate frame with the *xy*-plane determined by the HFC axes of the RP1 radicals. For the RP3 and RP4 models and their expansions, the angles (ϕ and θ) refer to the frame with the *z*-axis aligned to the EED axis. The symbols ●, ▲, and ✻ indicate, if relevant, the directions orthogonal to the FAD N5 HFC axis, the Trp N1 HFC axis, and the EED axis, respectively.

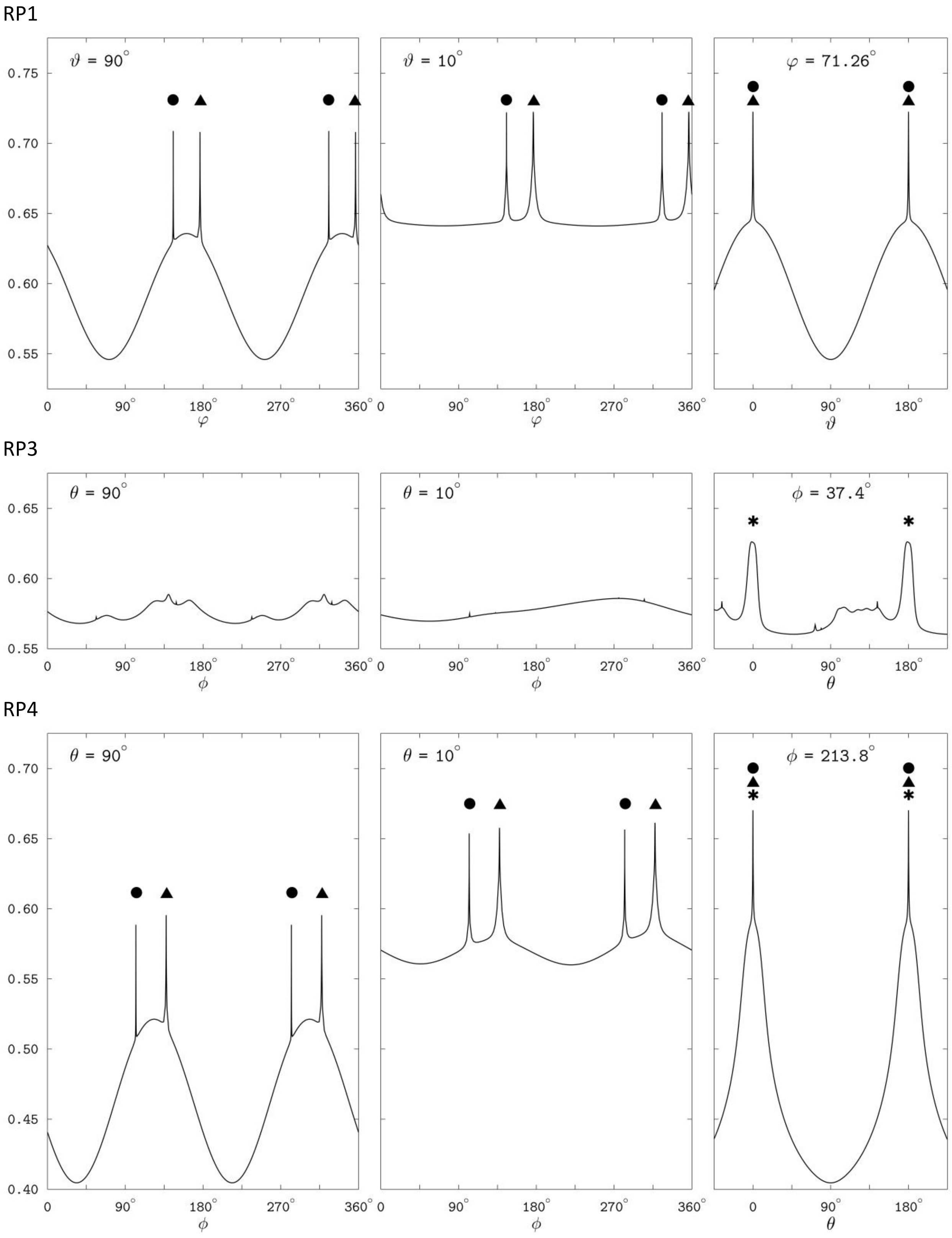

Figure S2. The interconversion triplet yield for RP1 (top row), RP3 (middle row), and RP4 (bottom row).

RP1a

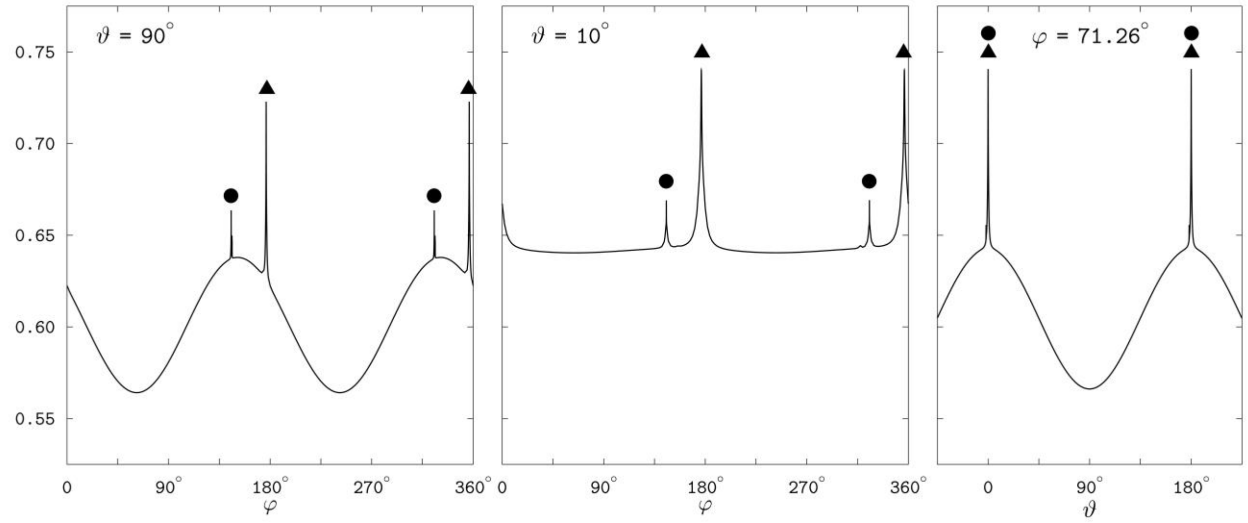

RP3a

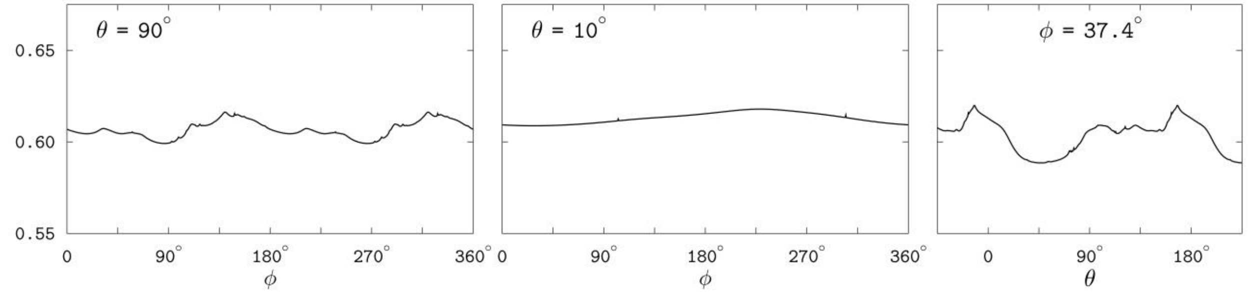

RP4a

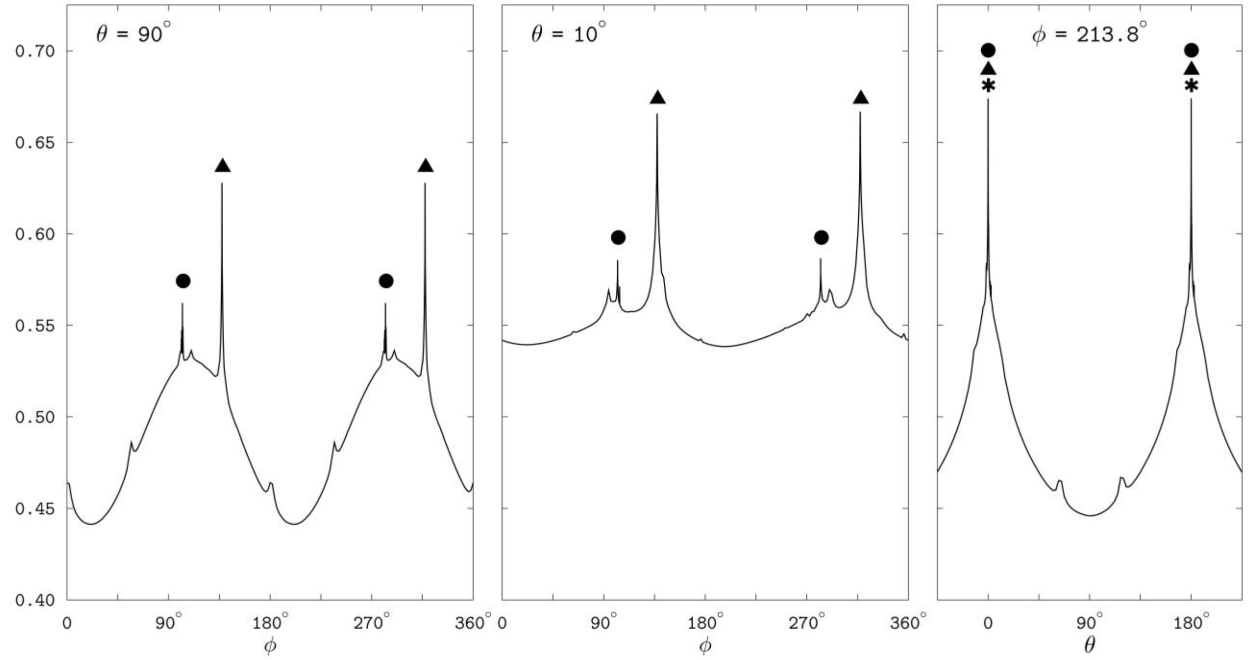

Figure S3. The interconversion triplet yield for RP1a, RP3a, and RP4a.

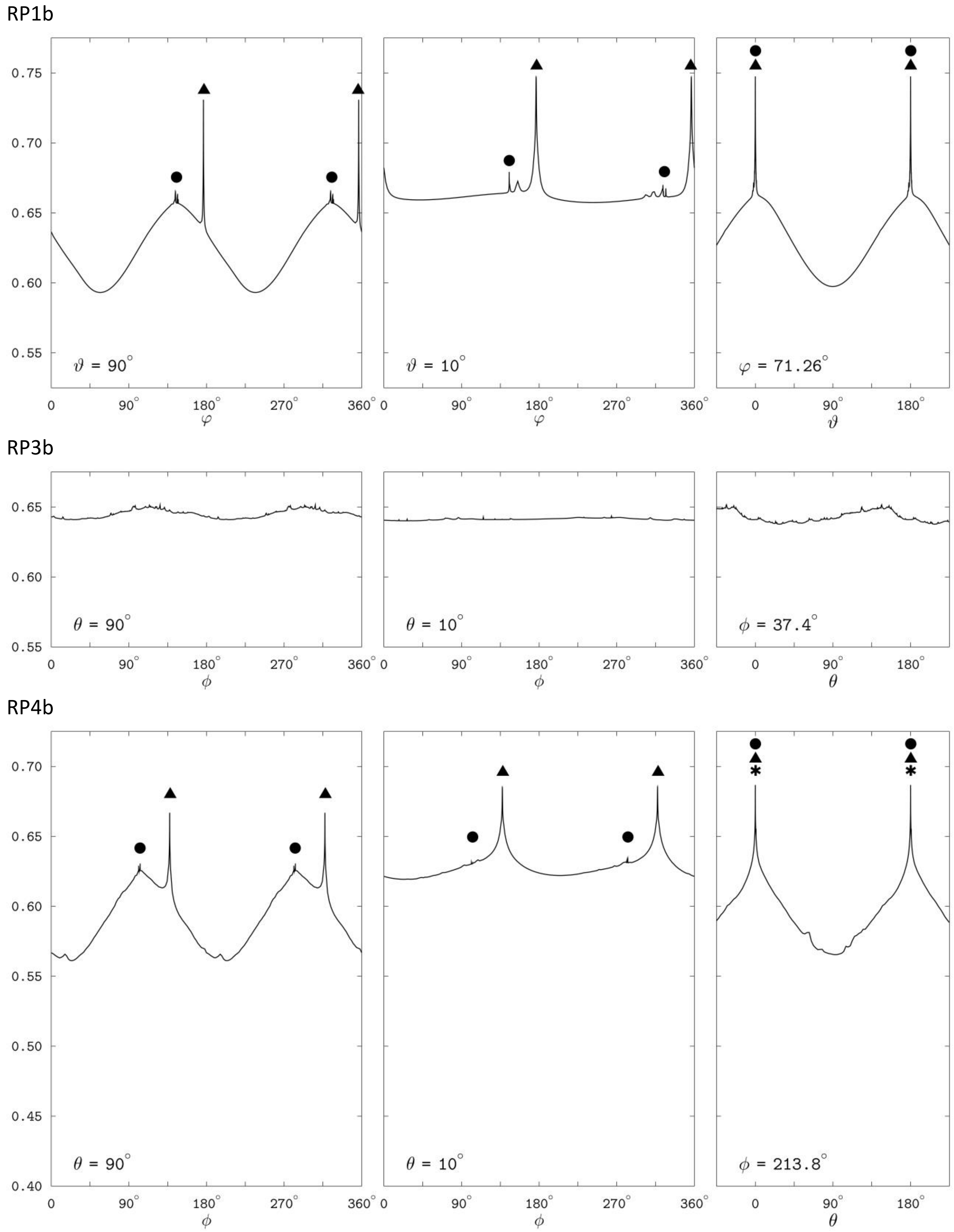

Figure S4. The interconversion triplet yield for RP1b, RP3b, and RP4b.

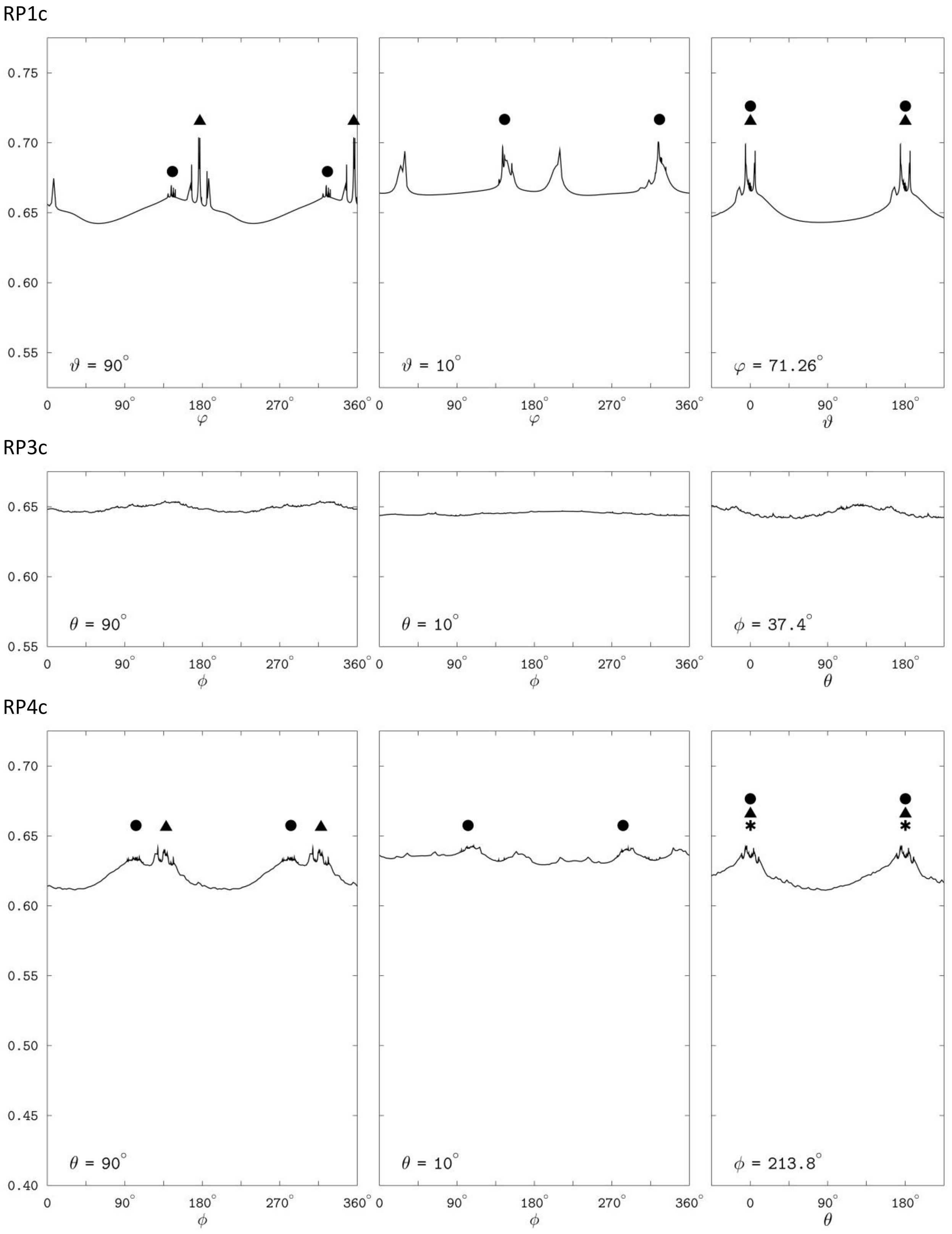

Figure S5. The interconversion triplet yield for RP1c, RP3c, and RP4c.

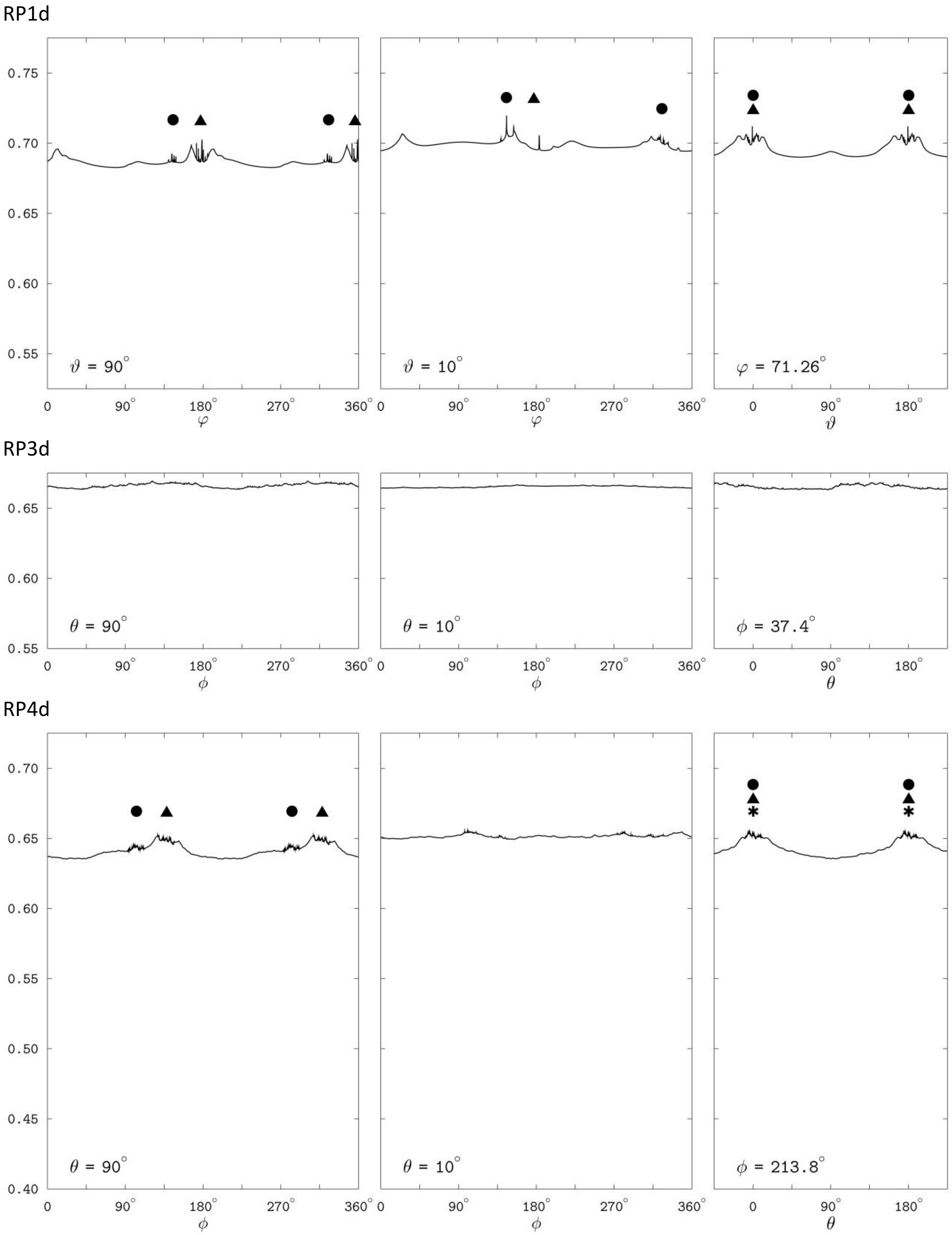

Figure S6. The interconversion triplet yield for RP1d, RP3d, and RP4d.
